# Bond Strength of Pretreated SFRC CAD/CAM Blocks: Comparison of Two SBS Test Methods

**DOI:** 10.3390/polym18080990

**Published:** 2026-04-19

**Authors:** Deniz Mizrak, Sufyan Garoushi, Pekka K. Vallittu, Mine Betul Uctasli, Lippo Lassila

**Affiliations:** 1 Department of Restorative Dentistry, Faculty of Dentistry, University of Cankiri Karatekin, 18100 Cankiri, Turkey; 2 Department of Biomaterials Science, Turku Clinical Biomaterial Center-TCBC, Institute of Dentistry, University of Turku, 20520 Turku, Finland; sufgar@utu.fi (S.G.); pekval@utu.fi (P.K.V.); liplas@utu.fi (L.L.); 3 Wellbeing Services County of Southwest Finland, 20521 Turku, Finland; 4 Department of Restorative Dentistry, Faculty of Dentistry, University of Gazi, 06490 Ankara, Turkey; uctasli@gazi.edu.tr

**Keywords:** CAD-CAM, composite resins, fiber-reinforced materials, shear strength, surface treatment

## Abstract

The reliability of adhesive bonding to CAD/CAM resin composites is influenced not only by material composition and surface treatment but also by the testing methodology used to assess bond strength. However, the impact of different shear bond strength (SBS) test configurations remains insufficiently clarified. This study evaluated the influence of different surface pretreatment protocols and SBS test methods on the bonding performance of a self-adhesive resin cement to two CAD/CAM materials: a conventional particulate-filled composite (Cerasmart 270) and an experimental short glass fiber-reinforced composite (SFRC CAD). Specimens (14 × 12 × 3 mm; *n* = 80 per material) were ground with 320-grit silicon carbide paper and divided according to surface pretreatment: airborne-particle abrasion (APA) or APA followed by hydrofluoric acid application for 60 s (APA + HF). Each group was further subdivided based on the SBS test method using either resin cement cylinders fabricated with a custom transparent mold (diameter: 3.6 mm; height: 3 mm) or metallic cylinders cemented to the treated surface. Half of the specimens were tested after 48 h of water storage, while the remainder underwent hydrothermal aging by boiling in water for 16 h prior to testing. Material type, SBS test method, surface pretreatment, and aging significantly affected bond strength (*p* < 0.05). The metallic cylinder method produced higher SBS values than the transparent mold technique, particularly for SFRC CAD. APA + HF tended to reduce SBS in Cerasmart 270, particularly after aging, whereas SFRC CAD showed comparable or higher bond strength values with APA alone. Aging decreased SBS in most groups. Overall, bond strength was influenced by both material type and test methodology. Within the limitations of this study, airborne-particle abrasion alone may be sufficient for SFRC CAD materials, while additional HF treatment may not provide further benefit. These findings highlight the importance of considering both material characteristics and test configuration when interpreting laboratory bond strength data.

## 1. Introduction

The increasing use of digital technologies in restorative dentistry has led to the widespread adoption of both subtractive (CAD/CAM) and additive manufacturing techniques. In particular, 3D-printed resin-based composites are emerging as promising alternatives, offering advantages such as reduced material waste and greater design flexibility [1]. However, CAD/CAM resin composites remain the most widely used materials in clinical practice due to their well-established mechanical properties and reliability [2]. Compared with conventional ceramics, CAD/CAM composites exhibit favorable esthetic properties, elastic moduli closer to those of dental tissues, and improved reparability, making them attractive alternatives for a wide range of clinical applications [2,3]. Conventional particulate-filled CAD/CAM composites are well established; [4,5] however, recent developments in fiber-reinforced composite technologies have prompted interest in the potential of short fiber-reinforced CAD/CAM composites to expand the indications for indirect composite restorations, despite their current experimental status [6,7].

Short fiber-reinforced composites (SFRCs) have been introduced to enhance the mechanical performance of resin-based materials by improving fracture toughness and inhibiting crack propagation [8,9]. The incorporation of short glass fibers within a resin matrix allows more effective stress distribution and energy dissipation, which may be particularly beneficial in high-load-bearing restorations [10]. With the introduction of SFRC materials in CAD/CAM block form, interest has grown in understanding their bonding behavior and long-term performance when luted with resin-based cements [7].

The longevity of indirect restorations is strongly dependent on the quality and durability of the adhesive bond between the CAD/CAM material and the resin cement [11]. This interface is affected by multiple factors, including material composition, surface pretreatment, and the type of adhesive system used [12,13]. Proper surface pretreatment is therefore essential to achieve reliable micromechanical interlocking and chemical adhesion. Airborne-particle abrasion (APA) is widely used to increase surface roughness and surface energy of CAD/CAM composites, whereas hydrofluoric acid (HF) etching has been shown to selectively dissolve glassy phases and enhance surface microroughness in silica-containing materials [14,15,16]. Nevertheless, the effectiveness of surface pretreatment protocols is highly material-dependent and influenced by factors such as filler composition, resin matrix structure, and the relative proportion of inorganic and organic phases [17].

In addition to material-related factors, the methodology used to evaluate bond strength plays a crucial role in the interpretation of results. Shear bond strength (SBS) testing remains one of the most commonly used laboratory methods for evaluating the adhesive performance of resin cements to restorative materials due to its simplicity and reproducibility. However, SBS test outcomes are highly sensitive to methodological variables, including specimen geometry, bonding area definition, loading configuration, and the mode of force application [18,19,20]. Moreover, SBS testing has been criticized for producing non-uniform stress distributions and incorporating tensile stress components at the adhesive interface, which may affect the reliability of the results. The use of microtensile bond strength (µTBS) testing could provide a more uniform stress distribution and a more reliable evaluation of the adhesive interface; however, this method is technique-sensitive and may lead to premature failure, particularly in brittle composite materials [21].

Furthermore, earlier investigations by our research group evaluated the bond strength and optical properties of SFRC CAD/CAM materials [7,22]. However, those studies did not specifically address the potential influence of different SBS test configurations on bond strength outcomes. As a result, the extent to which test methodology contributes to variability in measured bond strength values for SFRC CAD materials remains unclear.

In the transparent mold technique, resin cement cylinders are fabricated directly on the substrate, resulting in a relatively thicker cement layer through which shear force is applied. The increased cement thickness may influence stress distribution within the luting material during loading. In contrast, the metallic cylinder method involves bonding a prefabricated metal cylinder directly to the CAD/CAM surface, creating a thinner and more clinically relevant cement layer. During testing, the load is applied to a rigid metallic substrate, which may restrict deformation within the cement layer and enable a more controlled evaluation of the adhesive interface. Consequently, variations in cement thickness and load transfer mechanisms between different SBS techniques may influence the recorded bond strength values [19,20,23,24,25].

Therefore, the aim of the present study was to investigate the influence of shear bond strength (SBS) test methodology on the measured bond strength of resin cement to CAD/CAM composite materials. Specifically, the study compared two commonly used SBS test approaches—bonding of prefabricated metallic cylinders and the transparent mold technique—while also evaluating the effects of material type, surface pretreatment protocols, and hydrothermal aging on bond strength.

Based on the identified lack of consensus regarding the influence of test methodology and material structure on bond strength outcomes, the following null hypotheses were formulated:

(i) Surface pretreatment protocol would not significantly affect the SBS values regardless of material type.
(ii) The SBS test method would not significantly influence the measured bond strength values, irrespective of cement thickness and loading configuration.
(iii) Hydrothermal aging would not significantly affect SBS values, regardless of material type or test method.
(iv) No interaction effect would exist between the tested parameters.

## 2. Materials and Methods

### 2.1. Study Design and Materials

Table 1 presents the CAD/CAM materials and their compositions used in this study. A commercially available particulate-filled CAD/CAM composite (Cerasmart 270; GC Corp, Tokyo, Japan) and an experimental short glass fiber-reinforced composite (SFRC CAD) CAD/CAM block were investigated.

**Figure 1:**
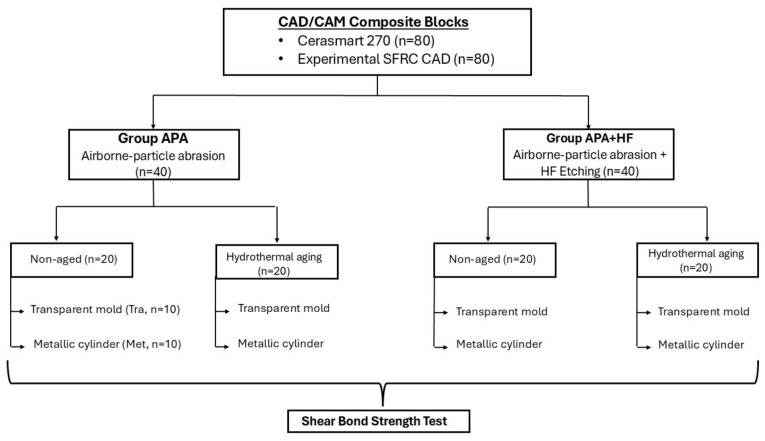
Schematic representation of the fully crossed factorial experimental design, illustrating the distribution of specimens according to material type, surface pretreatment, aging condition, and shear bond strength test method.

The experimental design consisted of a fully crossed 2 × 2 × 2 × 2 factorial structure, including material type, surface pretreatment, aging condition, and test method (Figure 1). Each subgroup included 10 specimens, resulting in a total sample size of 160.

The experimental SFRC CAD block was custom fabricated using a monomer matrix comprising 23 wt.%, which consisted of 70 wt.% urethane dimethacrylate (UDMA) and 30 wt.% triethylene glycol dimethacrylate (TEGDMA). The matrix was reinforced with 25 wt.% silanated short glass fibers (length: 200–300 µm; diameter: 6 µm) and 52 wt.% silanated barium glass fillers (mean particle size: 0.7 µm). The manufacturing process involved blending photoinitiators, fillers, fibers, and monomers into a homogeneous mixture using a Thinky Mixer ARV-310 (Thinky Corp., Tokyo, Japan). The resulting compound was placed into rectangular molds and subjected to compression in a pressure chamber to increase material density and prevent internal void formation. Polymerization was subsequently carried out under controlled high-temperature and high-pressure conditions, reaching temperatures of up to 170 °C to achieve optimal monomer conversion.

### 2.2. Specimen Preparation

Specimens were prepared from Cerasmart 270 and experimental SFRC CAD blocks by sectioning the blocks under continuous water cooling using a low-speed diamond saw (Secotom-50, Struers, Ballerup, Denmark). Rectangular specimens with dimensions of 14 mm × 12 mm × 3 mm were obtained from each material (*n* = 80 per material).

Each specimen was embedded in a cylindrical mold filled with autopolymerizing acrylic resin (Palapress Vario, Heraeus Kulzer, Hanau, Germany) to facilitate handling during testing. Bonding surfaces were standardized by sequential grinding with 180- and 320-grit silicon carbide papers using an automatic grinding machine (Rotopol-1, Struers, Denmark). Following grinding, specimens were thoroughly cleaned under running water and air-dried. Specimens were then randomly assigned to the experimental groups according to surface pretreatment and the SBS test method (Figure 1).

### 2.3. Surface Pretreatment

Specimens from each CAD/CAM material were divided into two surface pretreatment groups (*n* = 40 per group):

- Group APA: Airborne-particle abrasion only;
- Group APA + HF: Airborne-particle abrasion followed by hydrofluoric acid etching.

Airborne-particle abrasion was performed using 50 µm aluminum oxide particles (Korox, Bego, Bremen, Germany) with an intraoral airborne-particle abrasion device (CoJet System, 3M ESPE, St. Paul, MN, USA). The procedure was carried out at a distance of 10 mm, under a pressure of 2 bar, for 10 s, with the nozzle positioned perpendicular to the specimen surface.

For the APA + HF groups, specimens were rinsed with water spray after airborne-particle abrasion and subsequently etched with 4.5% hydrofluoric acid gel (IPS Ceramic Etching Gel, Ivoclar Vivadent AG, Schaan, Liechtenstein) for 60 s. After surface pretreatment, all specimens were thoroughly rinsed with water spray and air-dried.

### 2.4. Cement Application and Shear Bond Strength Testing

A self-adhesive resin cement (G-CEM One, GC Corp, Tokyo, Japan) was selected to maintain compatibility with the CAD/CAM composite materials and to follow the manufacturer’s recommended restorative system. The cement was mixed for 10 s according to the manufacturer’s instructions.

To evaluate the influence of the SBS test technique on bond strength outcomes, two different SBS test methods were employed: the transparent mold technique and a metallic cylinder bonding.

#### 2.4.1. Transparent Mold Technique (Tra)

**Figure 2:**
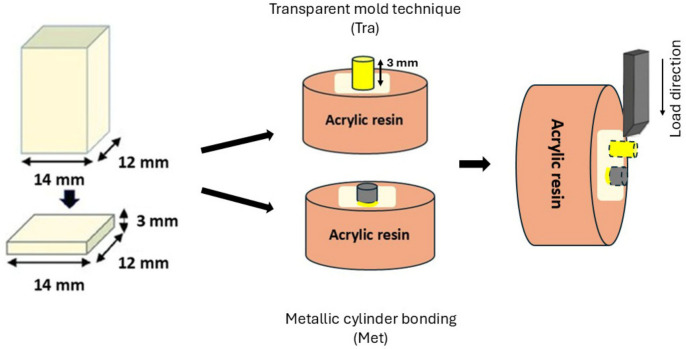
Schematic illustration of specimen preparation and shear bond strength (SBS) testing methods: the transparent mold technique (Tra), producing a 3 mm cement thickness using a standardized mold, and the metallic cylinder bonding (Met), resulting in a thin cement film. Load was applied perpendicular to the bonded interface.

A custom-made transparent mold with a flat end (diameter: 3.6 mm; height: 3 mm) was positioned centrally on the composite surface. The mold was filled with freshly mixed resin cement and light-polymerized through the transparent mold. After polymerization, the mold was carefully removed, resulting in the formation of a resin cement cylinder with a standardized height of 3 mm, ensured by the use of a standardized mold and bonded to the composite surface. During SBS testing, shear force was applied through the resin cement layer until failure occurred (Figure 2).

#### 2.4.2. Metallic Cylinder Bonding (Met)

Prefabricated metallic cylinders (3 mm in diameter and 3 mm in height) were centrally positioned on the flat composite surfaces. The self-adhesive resin cement was applied to bond the metallic cylinders directly to the CAD/CAM blocks, thereby standardizing both the bonding area. In this configuration, the cement layer formed as a thin film between the cylinder and the substrate, closely simulating the clinically relevant cement thickness observed during indirect restoration cementation procedures (Figure 2).

For both SBS test methods, excess cement was removed using a dental microbrush prior to polymerization. Light curing was performed for 20 s using an LED light-curing unit (Elipar S10, 3M ESPE, St. Paul, MN, USA). The light output (1592.7 mW/cm^2^ ± 5.3 mW/cm^2^) was monitored at the beginning of each group using a calibrated radiometer (MARC system, BlueLight Analytics, Halifax, NS, Canada).

### 2.5. Aging Protocol

Following cementation, specimens were divided into two aging conditions (*n* = 10 per subgroup). The non-aged group was stored in distilled water at 37 °C for 48 h. The aged group was subjected to hydrothermal accelerated aging by boiling in water for 16 h (UT6060, Heraeus Instruments, Hanau, Germany). This protocol was applied to promote water sorption and degradation of the resin matrix and fiber–matrix interface, allowing assessment of bond durability in both fiber-reinforced and non-fiber-reinforced CAD/CAM composites.

### 2.6. Shear Bond Strength Testing

Shear bond strength testing was performed using a universal testing machine (Lloyd LR30K Plus, Lloyd Instruments, Bognor Regis, UK) at a crosshead speed of 1.0 mm/min until failure.

### 2.7. Failure Mode Analysis

After SBS testing, failure modes were visually evaluated without magnification. Three independent and previously calibrated examiners assessed the failure modes according to standardized criteria. Failures were classified as adhesive (A), cohesive (C), or mixed (M).

### 2.8. Statistical Analysis

A posthoc power analysis was performed using G*Power software (version M10.1, Heinrich Heine University, Düsseldorf, Germany) to evaluate the adequacy of the sample size. Based on a medium-to-large effect size (f = 0.40), α = 0.05, and 16 groups, the calculated statistical power was approximately 0.90, indicating sufficient sensitivity to detect significant main and interaction effects.

SBS data were analyzed using a four-way analysis of variance (ANOVA) to evaluate the effects of material type (Cerasmart 270 vs. SFRC CAD), SBS test method (Tra vs. Met), surface pretreatment (APA vs. APA + HF), and aging condition (non-aged vs. aged), as well as their interactions. The assumptions of normality and homogeneity of variances were assessed using the Shapiro–Wilk test and Levene’s test, respectively. When significant differences were detected, post hoc comparisons were performed using Tukey’s HSD test. A correlation analysis (Spearman’s rho) was performed to evaluate the relationship between shear bond strength values and failure mode distribution. The level of statistical significance was set at α = 0.05.

## 3. Results

**Figure 3:**
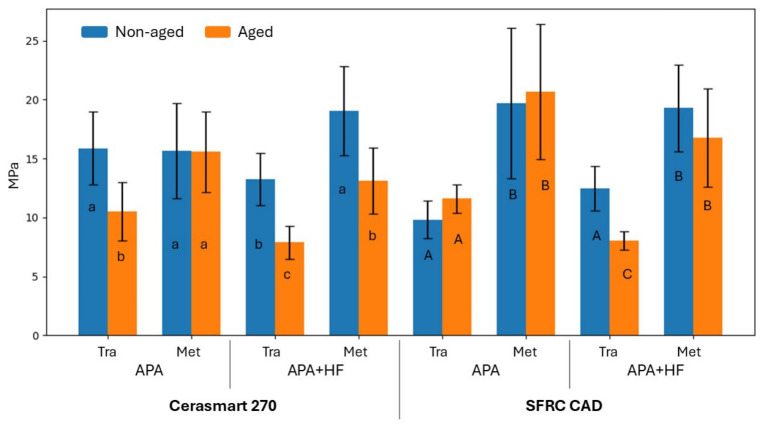
Shear bond strength (mean values ± standard deviations; MPa) of Cerasmart 270 and SFRC CAD composites obtained using two test methods (Tra: transparent mold; Met: metallic cylinder) after airborne-particle abrasion (APA) or APA followed by hydrofluoric acid etching (APA + HF), under non-aged and aged conditions. Different lowercase letters indicate statistically significant differences within Cerasmart 270, whereas uppercase letters indicate differences within SFRC CAD (*p* < 0.05).

Mean shear bond strength (SBS) values and standard deviations for all experimental groups are presented in Figure 3. The four-way ANOVA revealed that surface pretreatment (*p* = 0.0067), aging (*p* < 0.001), and test method (*p* < 0.001) had statistically significant effects on SBS values, whereas material type alone was not statistically significant (*p* = 0.147).

**Figure 4:**
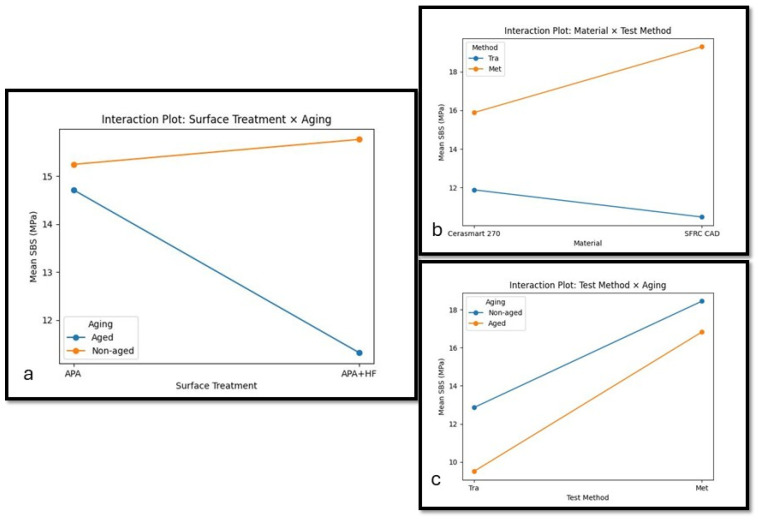
Interaction plots illustrate the combined effects of (**a**) surface pretreatment and aging condition, (**b**) material type and test method, and (**c**) test method and aging condition on shear bond strength (SBS) values (MPa).

However, despite the non-significant main effect of material, significant interaction effects were observed between material and aging (*p* = 0.004), surface treatment and aging (*p* < 0.001), material and test method (*p* < 0.001), and material × surface × method interaction (*p* = 0.034). These interaction effects are further illustrated in Figure 4. The four-way interaction was borderline significant (*p* = 0.050).

Effect size analysis (η^2^) indicated that the test method had the largest influence on SBS values (η^2^ = 0.39), followed by aging (η^2^ = 0.05) and material × method interaction (η^2^ = 0.05). Surface treatment showed a smaller but still notable effect (η^2^ = 0.02) (Table 2).

Overall, SBS values obtained using the metallic cylinder method (Met) were generally higher than those obtained with the transparent mold technique (Tra) for both CAD/CAM materials. This difference was more pronounced in the SFRC CAD groups, where the metallic cylinder method generally resulted in higher SBS values regardless of surface pretreatment and aging condition.

For Cerasmart 270, the application of APA + HF resulted in lower SBS values compared with APA alone in the Tra groups, particularly after aging. In contrast, when the Met method was used, APA and APA + HF produced comparable SBS values in the non-aged condition, while aged specimens showed a reduction in SBS following APA + HF.

For SFRC CAD, APA alone generally resulted in equal or higher SBS values than APA + HF, particularly when tested with the Met method. The reduction in SBS following APA + HF was more evident in the Tra groups, suggesting a material-dependent response to acid etching.

Hydrothermal aging had a material- and method-dependent effect on SBS values, rather than producing a consistent reduction across all groups. In Cerasmart 270, aging generally resulted in a decrease in SBS values, particularly in the Tra groups and in specimens treated with APA + HF. In contrast, the Met groups showed less pronounced changes after aging, especially in the APA condition. For SFRC CAD, aging had a variable effect on SBS, with some groups—particularly APA-treated specimens tested with the Met method—showing stable or slightly increased values, while APA + HF groups exhibited a reduction in SBS following aging. These findings indicate that the effect of aging should be interpreted in relation to baseline bond strength values and the specific test configuration.

Failure mode distributions are presented in Table 2. Representative images of adhesive, cohesive, and mixed failure modes are shown in Figure S1. In Cerasmart 270, failure modes varied according to surface pretreatment and SBS test method. Cohesive failures predominated in the APA groups, whereas APA + HF pretreatment resulted mainly in adhesive failures, particularly in the Tra groups, in both non-aged and aged conditions. The Met method was associated with a slightly higher incidence of cohesive failures compared with Tra, especially after APA. In contrast, SFRC CAD specimens consistently failed adhesively at the resin cement–composite interface across all surface pretreatments, test methods, and aging conditions, even when achieving high bond strength. No statistically significant correlation was found between shear bond strength values and failure mode distribution (Spearman’s ρ = 0.13, *p* = 0.107).

## 4. Discussion

The present study evaluated the influence of material type, surface pretreatment, aging, and most importantly, the shear bond strength (SBS) test method on the measured bond strength of a self-adhesive resin cement to CAD/CAM composites. The results demonstrated that, in addition to aging and material-related factors, the test methodology itself significantly affects bond strength outcomes. Therefore, all null hypotheses were rejected.

The longevity of indirect restorations largely depends on the integrity and durability of the adhesive interface between the CAD/CAM material and the luting cement [26,27]. Material type significantly affected SBS values, supporting previous reports indicating that adhesion to CAD/CAM composites is highly material-dependent [12,13,15]. The structural differences between particulate-filled composites and short fiber-reinforced composites (SFRCs) play a key role in stress distribution and interfacial behavior. The presence of short glass fibers within the resin matrix in SFRC materials contributes to improved fracture toughness and crack resistance, which may explain their relatively stable bonding performance even after aging [8,10].

Surface pretreatment significantly influenced bond strength, airborne-particle abrasion (APA) is one of the most widely recommended surface conditioning methods for CAD/CAM resin composites, as it increases surface roughness and surface energy, thereby enhancing micromechanical interlocking [27,28,29,30]. In the present study, APA alone provided stable and relatively high SBS values for SFRC CAD, even after hydrothermal aging, in agreement with previous reports demonstrating effective bonding of fiber-reinforced CAD/CAM composites without additional chemical treatment [7,31]

The effect of subsequent hydrofluoric acid (HF) etching was material-dependent. Although HF is effective in dissolving silica-based glass phases in ceramics [32], CAD/CAM resin composites exhibit a heterogeneous structure consisting of a polymer matrix and dispersed fillers, which may respond differently to acid treatment [33,34]. In Cerasmart 270, although APA + HF produced comparable or slightly higher initial bond strength in certain conditions (particularly with the Met method), aging resulted in a reduction in SBS values, consistent with hydrolytic degradation mechanisms reported for resin composites [35,36].

For SFRC CAD, APA + HF did not improve bond strength compared with APA alone. Given the fiber-reinforced microstructure of SFRC, which enhances stress distribution and crack resistance [7,37,38]. APA alone appears sufficient, and additional HF etching may be unnecessary or even disadvantageous. This may be attributed to the heterogeneous microstructure of fiber-reinforced CAD/CAM composites, where excessive etching may weaken the filler–matrix interface and increase susceptibility to hydrolytic degradation.

No untreated control group was included, as the study focused on clinically relevant pretreatment protocols. This may be considered a limitation when interpreting the absolute effect of surface treatments.

SBS testing is widely used due to its simplicity and reproducibility; however, it is highly sensitive to specimen geometry, load configuration, and stress distribution. Finite element analyses have demonstrated that conventional shear tests often generate non-uniform stress distributions with significant tensile components at the interface [23,24,25]. Alternative methods, such as microtensile bond strength (µTBS) testing, have been suggested to provide a more uniform stress distribution and higher sensitivity. Nevertheless, µTBS testing is more technique-sensitive and prone to specimen loss during preparation, limiting its applicability in multifactorial experimental designs [18,39]. Despite this, most studies have focused primarily on material properties and surface treatments, while the influence of SBS test methodology itself remains insufficiently explored [22]. In particular, there is limited evidence directly comparing different SBS test configurations under standardized conditions, making it unclear whether differences in measured bond strength values reflect true material performance or are primarily related to the testing methodology itself.

According to ISO/TS 11405, various specimen preparation methods can be used for SBS testing in dentistry, provided that the bonding area and testing conditions are clearly standardized and reported. The standard does not prescribe a specific specimen geometry, such as the use of metal cylinders or plastic molds, and both approaches are commonly reported in the literature [20,40]. However, ISO emphasizes the importance of controlling factors such as bonded area, cement thickness, specimen alignment, and loading configuration to maintain the reproducibility and comparability of bond strength measurements [41].

To the best of the authors’ knowledge, no previous study has systematically compared these two commonly used specimen preparation methods for measuring SBS, namely, bonding with rigid metal cylinders and the use of plastic or transparent molds to form the cement specimens. Therefore, a key aspect of the present study was the comparison of two SBS test methods: the transparent mold technique (Tra) and the metallic cylinder bonding method (Met).

One of the most important findings of this study is the significant influence of the SBS test method. The metallic cylinder method consistently yielded higher SBS values compared with the transparent mold technique, with a more pronounced effect observed in SFRC CAD. Effect size analysis further confirmed that the test method was the dominant factor influencing bond strength (η^2^ ≈ 0.39), exceeding the effects of material type and surface pretreatment. These findings are consistent with previous studies emphasizing that methodological variables can significantly influence bond strength outcomes [18,23]

This difference can be explained by variations in cement thickness and load transfer mechanisms between the two methods. In the transparent mold technique, a standardized mold results in a cement layer with a thickness of approximately 3 mm and a resin cement cylinder is formed directly on the substrate, and the applied load passes through the resin cement [7]. This configuration may introduce bending stresses within the cement layer, potentially leading to lower apparent SBS values [23].

In contrast, the metallic cylinder method produces a thinner cement layer, more closely resembling clinical cementation conditions and allows load transfer through a rigid component [22]. This configuration may reduce cement deformation and provide a more controlled evaluation of the adhesive interface. The significantly higher SBS values obtained with the metal cylinder method in the present study support the notion that methodological variables strongly influence bond strength measurements, as previously emphasized in macro bond test evaluations. Therefore, the measured SBS values should be interpreted not only as intrinsic material properties but also as outcomes influenced by the testing methodology. This highlights the importance of considering test configuration when comparing bond strength data across studies [20,23].

In addition to cement thickness, the mismatch in elastic modulus between the resin cement, composite substrate, and loading assembly may further influence stress distribution at the bonded interface. In the transparent mold technique, the relatively thick and more compliant resin cement layer may undergo greater deformation under load, leading to stress concentration within the cement and at the interface. In contrast, in the metallic cylinder method, the rigid metal component transfers the applied load more directly to the interface, reducing bulk deformation within the cement layer. This difference in load transfer mechanism may contribute to the higher and more stable SBS values observed with the metallic cylinder method. These findings are consistent with previous finite element studies demonstrating that stress distribution in bonded interfaces is highly influenced by cement thickness, elastic modulus mismatch, and loading configuration [18,23,42]

Hydrothermal aging influenced bond strength in a material- and pretreatment-dependent manner. In Cerasmart 270, aging resulted in a noticeable reduction in SBS values, particularly in the APA + HF groups. In contrast, SFRC CAD demonstrated greater resistance to aging, with stable or slightly increased values in APA-treated groups, especially when tested using the metallic cylinder method (Figure 3).

The use of boiling water for hydrothermal aging represents an accelerated aging approach rather than a direct simulation of intraoral conditions. Nevertheless, it is widely accepted as an effective method for inducing hydrolytic degradation in resin-based materials within a shorter experimental time frame [35,36]. Previous studies have demonstrated that boiling in water promotes water diffusion into the polymer matrix, leading to plasticization and softening of the resin structure, particularly at the filler–matrix interface [7,29,30]. Furthermore, water infiltration may contribute to the hydrolysis of silane coupling agents, thereby weakening the chemical bonding between the resin matrix and inorganic fillers [35]. This approach enables accelerated hydrolytic degradation and allows comparison of material behavior under more aggressive conditions.

Failure mode analysis revealed predominantly adhesive failures, particularly in SFRC CAD groups, at the cement–composite interface, even at higher bond strength levels (Table 3). The fiber-reinforced structure enhances fracture toughness and resistance to crack propagation within the substrate, thereby shifting the weakest link of the bonded assembly to the adhesive interface. Consequently, failure is more likely to occur at the interface rather than within the material itself [8,37,38]. This finding is consistent with previous studies on SFRC CAD materials, where predominantly adhesive failures have been reported despite relatively high bond strength values [7,22].

In contrast, Cerasmart 270 demonstrated a higher incidence of cohesive or mixed failures in high-strength groups, indicating that substrate properties may become the limiting factor once sufficient interfacial adhesion is achieved. Although failure mode analysis provides qualitative insight into the nature of bond failure, a direct correlation between failure mode distribution and bond strength values is not always observed. In the present study, no statistically significant correlation was found, indicating that failure patterns may be influenced by multiple factors and do not necessarily reflect bond strength values alone (Spearman’s ρ = 0.13, *p* = 0.107).

In recent years, 3D-printed resin-based composites have emerged as promising alternatives in digital dentistry, offering advantages such as customized fabrication and reduced material waste. However, their microstructure and interlayer bonding characteristics differ significantly from those of CAD/CAM composites, which may influence their bonding behavior [1,43,44]. Although the present study focused on milled CAD/CAM materials, future investigations should include 3D-printed composites to better reflect current trends and to determine whether similar trends in test methodology and surface treatment are observed in additively manufactured systems.

This study has certain limitations that should be considered when interpreting the results. Only one type of self-adhesive resin cement and two CAD/CAM composite materials were evaluated, which may limit the generalizability of the findings. In addition, surface characterization techniques such as scanning electron microscopy (SEM) were not performed, limiting the ability to correlate surface morphology with bonding performance.

Furthermore, although SBS testing provides valuable comparative data, alternative methods such as microtensile bond strength (µTBS) testing could offer a more detailed assessment of the adhesive interface. Additionally, the aging process simulated hydrothermal accelerated degradation but did not account for other intraoral factors such as pH fluctuations, mechanical loading, long-term water storage, or repeated thermal cycling. Therefore, further studies incorporating a wider range of materials, emerging technologies such as 3D-printed composites, advanced surface characterization methods, and more clinically relevant aging protocols are needed to confirm and extend these findings.

## 5. Conclusions

From a clinical perspective, the findings suggest that airborne-particle abrasion alone may be sufficient for bonding to SFRC CAD materials, whereas conventional particulate-filled CAD/CAM composites may be more sensitive to surface pretreatment protocols and aging conditions.

Within the limitations of this study, shear bond strength (SBS) values were significantly influenced not only by material type, surface pretreatment, and aging, but also by the test method itself. The metallic cylinder method yielded higher SBS values, likely due to differences in cement thickness and load transfer mechanisms. Clinically, bonding configurations that produce thinner cement layers may better reflect real conditions. Therefore, the test methodology should be carefully considered when interpreting and comparing bond strength data across studies.

## Figures and Tables

**Table 1:** CAD/CAM composites and luting resin used in the study.

Material	Manufacturer	Composition
Cerasmart 270	GC Corp, Tokyo, Japan	Bis-MEPP, UDMA, dimethacrylate, Silica (20 nm), barium glass (300 nm, 71 wt.%)
CAD/CAM Short Fiber-Reinforced Composite Block	Experimental	UDMA, TEGDMA, short glass fiber (200–300 µm & Ø6 μm), barium glass (77 wt.%)
G-CEM ONE Universal self-adhesive resin cement	GC Corp, Tokyo, Japan	Paste A: Fluoroaluminosilicate glass, titanium dioxide, UDMA, 2-Hydroxy-1,3 dimethacryloxypropane, initiators Paste B: Silica filler, UDMA, 10-MDP, dimethylbenzyl hydroperoxide.

TEGDMA, triethylene glycol dimethacrylate; UDMA, urethane dimethacrylate; Bis-MEPP, Bis (p-methacryloxy (ethoxy) 1-2 phenyl)-propane; 10-MDP, 10-methacryloyloxydecyl dihydrogenphosphate; wt.%, weight percentage.

**Table 2:** Results of the four-way ANOVA showing the effects of material type, surface pretreatment, aging condition, and test method on shear bond strength (SBS), including *p*-values and effect sizes (η^2^).

Factor/Interaction	*p*-Value	Effect Size (η^2^)
Material	0.147	0.006
Surface pretreatment	0.0067	0.021
Aging	<0.001	0.052
Test method	<0.001	0.393
Material × Surface	0.966	<0.001
Material × Aging	0.004	0.023
Surface × Aging	<0.001	0.043
Material × Method	<0.001	0.050
Surface × Method	0.984	<0.001
Aging × Method	0.066	0.009
Material × Surface × Aging	0.335	0.003
Material × Surface × Method	0.034	0.013
Material × Aging × Method	0.393	0.002
Surface × Aging × Method	0.253	0.004
Material × Surface × Aging × Method	0.050	0.011

**Table 3:** Distribution of failure modes according to material type (Cerasmart 270 and SFRC CAD), surface pretreatment (APA and APA + HF), SBS test method (Tra: Transparent mold; Met: metallic cylinder), and aging condition (A: Adhesive, C: Cohesive, M: Mix).

Material	Surface Treatment	SBS Test Method	Non-Aged			Aged		
			A	C	M	A	C	M
	APA	Tra	0	10	0	0	10	0
Cerasmart 270		Met	2	8	0	0	10	0
	APA + HF	Tra	8	1	1	9	1	0
		Met	6	4	0	8	2	0
	APA	Tra	10	0	0	10	0	0
SFRC CAD		Met	10	0	0	10	0	0
	APA + HF	Tra	10	0	0	10	0	0
		Met	10	0	0	10	0	0

## Data Availability

The original contributions presented in the study are included in the article; further inquiries can be directed to the corresponding author.

## Supplementary Materials

The following supporting information can be downloaded at: https://www.mdpi.com/article/10.3390/polym18080990/s1. Figure S1: Representative failure modes after shear bond strength (SBS) testing.
