# Supplementary figures and images for: Bond Strength of Pretreated SFRC CAD/CAM Blocks: Comparison of Two SBS Test Methods
Source: Polymers (Basel). 2026 Apr 19;18(8):990. doi: 10.3390/polym18080990 (PMC13120386; doi:10.3390/polym18080990)

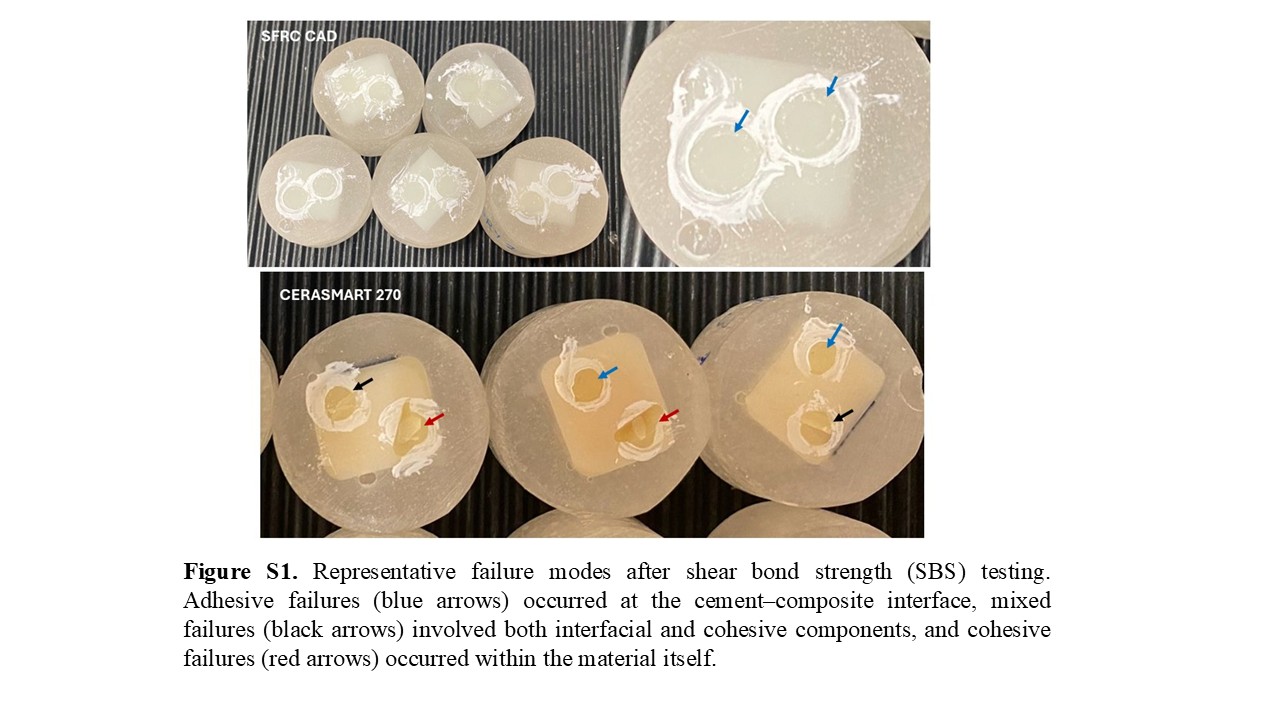

Supplement: Supplementary file 1 [file polymers-18-00990-s001.zip › fig. s1.jpg]
